# Mitotic MTH1 inhibitor karonudib kills epithelial ovarian cancer independent of platinum sensitivity

**DOI:** 10.1186/s40164-025-00681-0

**Published:** 2025-06-23

**Authors:** Rachel M. Hurley, Jill M. Wagner, Arun Kanakkanthara, Annapoorna Venkatachalam, Aaron M. Deisinger, Cristina Correia, Paula A. Schneider, Kevin L. Peterson, Elaine P. Macon, Ethan P. Heinzen, Kumar Sanjiv, Xiaonan Hou, Marc A. Becker, Matthew J. Maurer, Melissa C. Larson, Elizabeth M. Swisher, Hu Li, Ann L. Oberg, S. John Weroha, Ulrika Warpman Berglund, Thomas Helleday, Scott H. Kaufmann, Andrea E. Wahner Hendrickson

**Affiliations:** 1 Department of Molecular Pharmacology & Experimental Therapeutics, Mayo Clinic, 55905 Rochester, MN USA; 2 Department of Oncology, Mayo Clinic, MN 55905 Rochester, USA; 3 Division of Clinical Trials and Biostatistics, Department of Quantitative Health Sciences, Mayo Clinic, MN 55905 Rochester, USA; 4 Robert D. And Patricia E. Kern Center for the Science of Health Care Delivery, Mayo Clinic, MN 55905 Rochester, USA; 5 Science for Life Laboratory, Division of Translational Medicine and Chemical Biology, Department of Oncology and Pathology, Karolinska Institutet, 171 65 Stockholm, Sweden; 6 Department of Obstetrics and Gynecology, University of Washington, WA 98195 Seattle, USA; 7 Division of Computational Biology, Department of Quantitative Health Sciences, Mayo Clinic, Rochester, MN USA; 8 Oxcia AB, 113 34 Stockholm, Sweden

**Keywords:** MTH1 inhibitors, Ovarian cancer, Platinum resistance, PDX models

## Abstract

The prognosis for women with ovarian cancer (OC) is particularly poor if resistance to platinum compounds, the mainstay of standard-of-care therapy, develops. Inhibitors of the Nudix hydrolase MuT Homolog 1 (MTH1) have previously been shown to arrest cancer cells in mitosis, increase 8-oxo-2’-deoxyguanosine (8-oxo-dG) incorporation into DNA, and selectively kill neoplastic cells while sparing normal cells. Here we explored the cytotoxic mechanism of these agents as well as their activity against platinum-resistant OC in vitro and in vivo. Two mitotic MTH1 inhibitors (mMTH1is), TH588 and karonudib, decreased colony formation indistinguishably in platinum-sensitive OC cell lines and their platinum-resistant counterparts in vitro but had limited effects on fallopian tube and immortalized ovarian surface epithelial cells. Treatment with karonudib stalled OC cells in mitosis and caused elevated 8-oxo-dG levels in DNA followed by activation of base excision repair, induction of BAX, and apoptotic cellular demise. This cytotoxicity was blunted by overexpression of the pre-mitotic checkpoint protein CHFR, which inhibits other anti-mitotics, or treatment with the antioxidant N-acetylcysteine, which diminishes nuclear 8-oxo-dG staining, suggesting a role for both mitotic stalling and increased nuclear incorporation of oxidized nucleotides in karonudib efficacy. In three orthotopic OC patient-derived xenograft models, karonudib monotherapy induced growth delay in vivo. Moreover, addition of karonudib to carboplatin doubled median overall survival in two models and prolonged survival for the duration of the study (110 days) in the third. These results demonstrate activity of mMTH1is as monotherapy and in combination with carboplatin in OC that warrants further investigation.

**Supplementary Information:**

The online version contains supplementary material available at 10.1186/s40164-025-00681-0.

To the Editor,

Resistance to platinum compounds, the mainstay of standard-of-care ovarian cancer (OC) therapy [1, 2], develops in a majority of cases and is associated with a one-year median survival [3, 4], highlighting the need for new therapies. The Nudix hydrolase MutT Homolog 1 (MTH1) is elevated in various cancers [5] and required for survival of cells transformed by a number of oncogenes [6], but not normal cells, leading to efforts to inhibit this enzyme [7, 8]. Recent studies demonstrated that some MTH1 inhibitors concomitantly arrest neoplastic cells in mitosis, reflecting a previously unappreciated role of MTH1 in cell division, and increase 8-oxo-2’-deoxyguanosine (8-oxo-dG) incorporation into DNA [8, 9]. Here we explored the activity of these mitotic MTH1 inhibitors (mMTH1is) against platinum-resistant OC in vitro and in vivo.

To assess antiproliferative activity, OC cell lines (Supplementary Table S1) were treated with the first-generation mMTH1i TH588 (Supplementary Fig. S1A) or second-generation inhibitor karonudib (Fig. 1A) in colony forming assays (**Supplementary Methods**). All lines were sensitive, with IC_50_s of 0.9-4 µM for TH588 and 60-200 nM for karonudib. Mechanistic studies in the A2780 cells indicated these effects on colony formation reflected a process that involved incorporation of 8-oxo-dG into DNA (see below), activation of base excision repair as manifested by XRCC1 foci, activation of DNA damage-activated phosphatidylinositol-3 kinase-related kinases (γH2Ax foci), TP53 accumulation, increased expression of *TP53* target genes, increased levels and mitochondrial translocation of the proapoptotic protein BAX, mitochondrial outer membrane depolarization, and development of apoptotic morphological changes in live cell imaging (Figs. S2 and S3). In contrast, nontransformed immortalized ovarian surface epithelial (IOSE) and fallopian tube epithelial cells demonstrated minimal response (Fig. 1B, S1B and S1C).

**Fig. 1:**
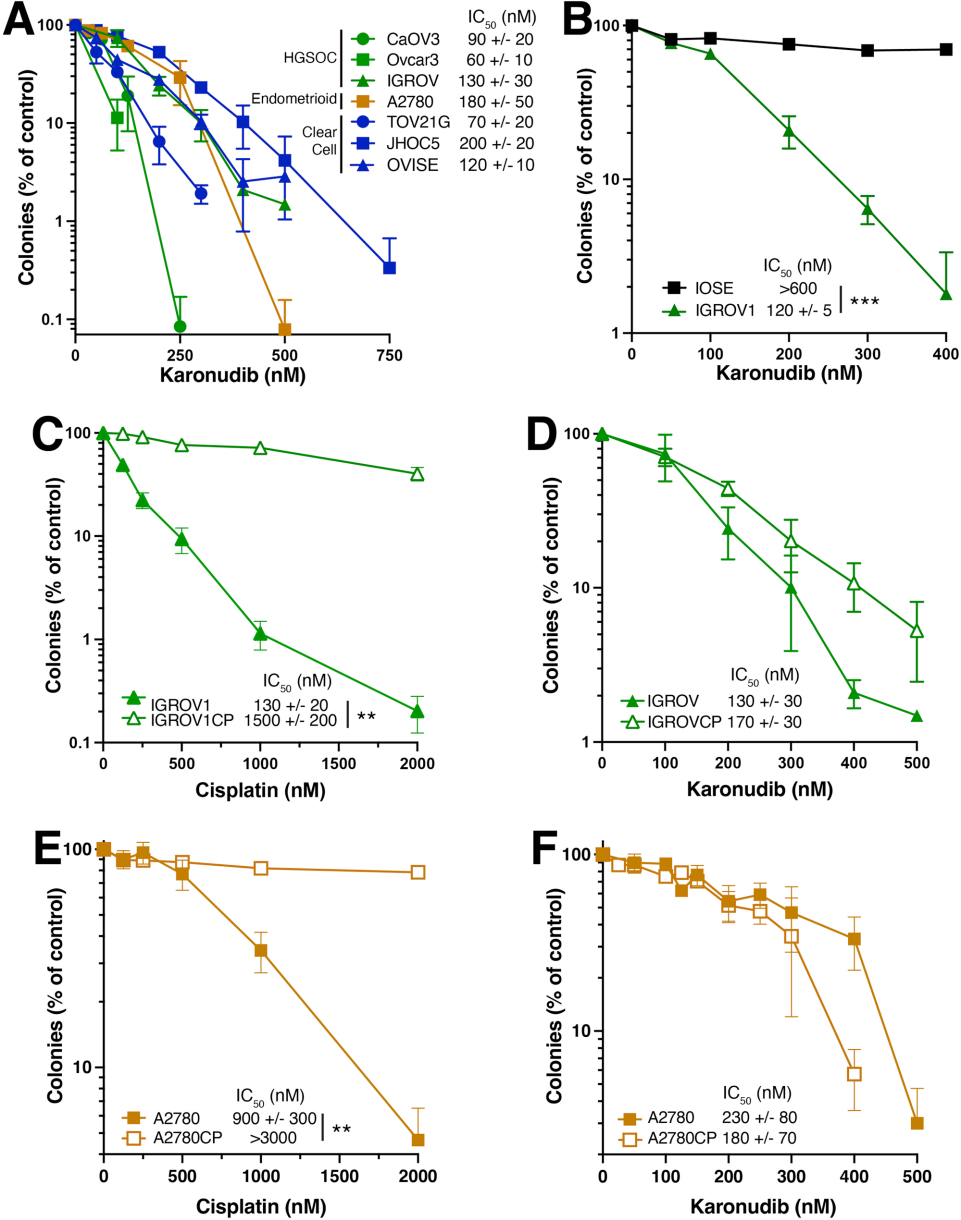
mMTH1 inhibitor sensitivity in platinum-sensitive and -resistant OC cell lines. **A, B**, cell lines corresponding to the indicated OC histological subtypes (**A**) or IGROV1 and immortalized normal ovarian surface epithelial cells (IOSE, **B**) were treated continuously with the mMTH1i karonudib in clonogenic assays. **C-F**, paired parental IGROV1 and platinum-selected IGROV1/CP cells (**C**, **D**) or A2780 and platinum-selected A2780/CP200 cells (**E**, **F**) were treated continuously with the indicated concentrations of cisplatin (**C**, **E**) or karonudib (**D**, **F**). Numbers indicate IC_50_ values (mean ± sd) from 2 (OVISE) or ≥3 independent replicates (all other lines). IC_50_ values for IGROV1 and A2780 cells vary slightly between panels because resistant lines were directly compared to parental lines, which were repeated for those experiments. *, ** and *** indicate *p* < 0.05, 0.01 and 0.001 by 2-sided *t* tests, respectively. *t* tests comparing IC_50_s in panels D and F were not significant. Assays of TH588 sensitivity are presented in Fig. S1

To determine whether mMTH1is also demonstrate efficacy in platinum-resistant OC lines, paired parental and cisplatin-resistant cell lines were compared. Despite a >10-fold difference in cisplatin IC_50_s (Fig. 1C), responses of parental IGROV and cisplatin-resistant IGROV1/CP cells to mMTHis were similar (Fig. 1D, S1D). Comparable results were observed when parental A2780 and PEO1 cells were compared to their respective platinum-resistant counterparts (Fig. 1E, 1 F, S1E-G).

Previous studies have demonstrated two actions of karonudib: (i) stalling of cells in mitosis and (ii) accumulation of oxidized nucleotides, including 8-oxo-dGTP, into DNA [9]. To assess the contributions of these effects to karonudib activity in OC, MTH1i-treated cells were examined for mitotic stalling by flow cytometry (Fig. S4A-S4C) or morphological examination (Fig. S4D, S4E). Both parental and platinum-resistant A2780 cells were transiently stalled in mitosis, although not to the same extent as by paclitaxel (Fig. S4A-S4E). The potential impact of this mitotic stalling was assessed by comparing the action of MTHis in A2780 cells that differ in expression of CHFR, a premitotic checkpoint protein that imparts the ability of cells to arrest prior to mitosis and avoid mitotic catastrophe [10, 11]. A2780 clones expressing CHFR (Fig. S4F) had decreased sensitivity to karonudib as well as paclitaxel (Figs. S4G, S4H), suggesting an important role for mitotic arrest in karonudib-induced killing.

To assess the role of increased nucleotide oxidation in mMTH1i-induced killing, we examined the impact of the antioxidant N-acetylcysteine (NAC) on nuclear 8-oxo-dG levels and clonogenic survival. NAC pretreatment blunted the mMTH1i-induced increase in 8-oxo-dG fluorescence (Fig. S5A-D) and diminished the impact of karonudib on colony formation (Fig. S5E, S5F), supporting a role for 8-oxo-dG incorporation in mMTH1i-induced killing as well.

Based on this role of oxidative damage in mMTH1i-induced killing and previous reports indicating that platinum agents increase cellular reactive oxygen species [12–14], we assessed activity of the karonudib/carboplatin combination in vivo. For these studies, three genomically distinct high grade serous OC patient-derived xenograft (PDX) models (Table S1) with varying platinum sensitivity were implanted orthotopically, allowed to engraft, and monitored by transabdominal ultrasound during and after treatment with diluent, karonudib, carboplatin, or the combination.

Karonudib enhanced the effects of carboplatin in all three models (Figs. 2 and S6) without enhancing toxicity at the final dose and schedule (Fig. S7). In PH384, a platinum tolerant model harboring *TP53* and *LIG4* mutations, the karonudib/carboplatin combination induced tumor regressions (Fig. 2A, *p* < 0.0001 relative to control) and increased overall survival relative to platinum monotherapy [Hazard ratio (HR) for combination versus carboplatin monotherapy 0.22 (0.05–0.91), *p* = 0.037, Fig. 2B]. Moreover, karonudib also increased nuclear 8-oxo-dG in vivo (Fig. 2C), as anticipated from the cell line studies. In the highly platinum sensitive PH013 model, karonudib induced tumor shrinkage, providing the first evidence for regressions induced by karonudib monotherapy in a carcinoma model (Fig. 2D). In addition, there was a trend toward increased survival in the combination arm relative to carboplatin (Fig. 2E) that did not reach statistical significance due to the small number of events. In PH450, a *CHEK2* mutated model that is highly platinum-resistant, addition of karonudib enhanced carboplatin-induced slowing (Fig. 2F, *p* = 0.002 for combination vs. carboplatin) and increased overall survival (Fig. 2G) [HR for combination vs. carboplatin 0.27 (0.08–0.88), *p* = 0.03] while also increasing levels of nuclear 8-oxo-dG relative to the increase with carboplatin alone (Fig. 2H).

**Fig. 2:**
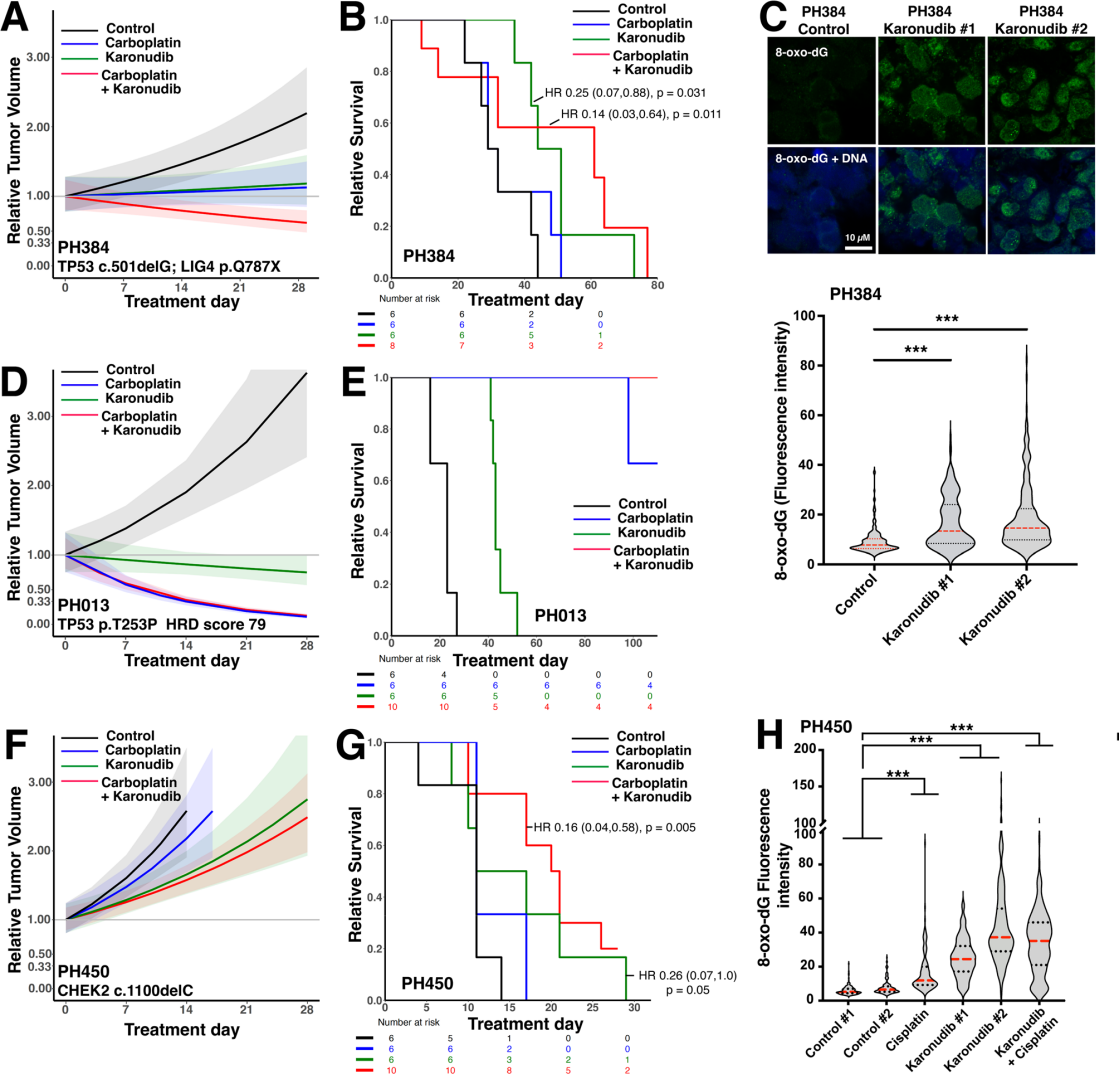
Effects of karonudib as monotherapy and in combination with carboplatin in orthotopic high grade serous ovarian cancer PDXs. Karonudib was administered at 90 mg/kg as a single agent (all PDXs) or in combination with carboplatin (50 mg/kg/week) at karonudib doses of 90 mg/kg (**A-C**) or 60 mg/kg (**D-H**) to mice bearing intraperitoneal high grade serous ovarian cancer PDX models PH384 (**A-C**), PH013 (**D-E**), or PH450 (**F-H**). PDX growth was measured by transabdominal ultra-sound and expressed as cross-sectional area relative to Day 0 (**A**, **D**, **F**). Error bars indicate 95% confidence intervals. Following 28 days of treatment, tumor growth and overall survival (**B**, **E**, **G**) were observed for up to 110 days. Numbers indicate hazard ratios (95% confidence intervals) and corresponding *p* values for indicated treatments relative to controls. In addition, FFPE samples harvested on day 5 of treatment from PH384 and PH450 were stained for nuclear 8-oxo-dG (e.g., micrographs at top of panel C), with fluorescence intensity (**C**, **H**) determined as indicated in the Methods. Fluorescence of 100–250 individual nuclei/sample is indicated. Lines in panels C and H indicate median (red) and interquartile values (black). *** indicates *p* < 0.001 by Wilcoxon rank sum test after correction for multiple comparisons. Growth curves of PDXs in individual mice are shown in Fig. S6

Collectively, these results demonstrate that mMTH1is exhibit indistinguishable antiproliferative effects in paired platinum sensitive and platinum resistant OC lines in vitro, that both mitotic stalling and incorporation of oxidized nucleotides into DNA are important for these antiproliferative effects, and that antineoplastic effects of carboplatin in three OC PDX models are enhanced by the clinical mMTH1i karonudib in vivo. In view of the safety and monotherapy activity observed in the recently completed karonudib phase I trial (NCT03036228, ref. 15), further study of the karonudib/carboplatin combination might be warranted, providing a potential path toward clinical development of karonudib for OC.

## Electronic supplementary material

Below is the link to the electronic supplementary material.

Supplementary Material 1

## Data Availability

RNAseq data (Figure S2) are deposited in the Gene Expression Omnibus database (GSE293549). Other datasets generated during the current study are available from the corresponding authors on reasonable request.

## Abbreviations

8-oxo-dG: 8-oxo-2’deoxyguanosine
FBS: Heat-inactivated fetal bovine serum
HGSOC: High grade serous ovarian cancer
HR: Hazard ratio
MTH1: MutT Homolog 1
MTH1i: MTH1 inhibitor
mMTH1i: Mitotic MTH1 inhibitor
NAC: N-acetylcysteine
OC: Ovarian cancer
PBS: Calcium- and magnesium-free Dulbecco’s phosphate buffered saline
PDX: Patient-derived xenograft
RFT: Resected fallopian tube
ROS: Reactive oxygen species
