# Supplementary material for: Mitotic MTH1 inhibitor karonudib kills epithelial ovarian cancer independent of platinum sensitivity
Source: Exp Hematol Oncol. 2025 Jun 23;14:88. doi: 10.1186/s40164-025-00681-0 (PMC12183885; doi:10.1186/s40164-025-00681-0)
Supplement: Supplementary file 1 — Supplementary Material 1 [file 40164_2025_681_MOESM1_ESM.pdf]

Supplementary Material for

MTH1 Inhibitor Karunodib Kills Epithelial Ovarian Cancer Independent of Platinum Sensitivity

Rachel M. Hurley, Jill M. Wagner, Arun Kanakkanthara, Annapoorna Venkatachalam, Aaron M. Deisinger, Cristina Correia, Paula A. Schneider, Kevin L. Peterson, Elaine P. Macon, Ethan P. Heinzen, Kumar Sanjiv, Xiaonan Hou, Marc A. Becker, Matthew J. Mauer, Melissa C. Larson, Elizabeth M. Swisher, Hu Li, Ann L. Oberg, S. John Weroha, Ulrika Warpman Berglund, Thomas Helleday, Scott H. Kaufmann, and Andrea E. Wahner Hendrickson

SUPPLEMENTARY METHODS

Materials

Ovarian cancer (OC) cell lines and patient-derived xenografts (PDXs) were obtained as indicated in **Supplementary Table S1**. TH588 and karonudib were synthesized as previously described (1, 2). All other reagents and antibodies were obtained as described in **Table S2**.

Cell Lines

The following OC cell lines were utilized: IGROV1, IGROV1/CP and OVISE from Dr. Ernst Lengyel (University of Chicago, IL); OVCAR3 and TOV21G cells from American Type Culture Collection (Manassas, VA); CaOV3 and immortalized ovarian surface epithelial cells (IOSE) from Viji Shridhar (Mayo Clinic, Rochester, MN); A2780 and A2780/CP200 from Thomas Hamilton (Fox Chase Cancer Center, Philadelphia, PA); PEO1 cells from Fergus Couch (Mayo Clinic, Rochester, MN); PEO1 C4-2 cells from Toshiyasu Tanaguchi (Fred Hutchinson Cancer Center, Seattle, WA), and JHOC5 from Evette Radisky (Mayo Clinic, Jacksonville, FL). Genomic features of these lines are summarized in **Table S1**. All cell lines were authenticated by short tandem repeat analysis in the Mayo Clinic Cytogenetics Core Facility and assayed regularly for mycoplasma. A2780 clones expressing S peptide-tagged CHFR were generated as previously described (3). Resected fallopian tube (RFT) cells were derived by culture of epithelial cells recovered from fimbria resected at the University of Washington for non-neoplastic indications in accordance with an institutional review board-approved protocol.

Cells were grown in the following media: IGROV1 and IGROV1/CP cell lines in Medium A consisting of RPMI 1640 medium with 10% (vol/vol) heat-inactivated fetal bovine serum (FBS); OVCAR3 as well as A2780 and its derivatives in Medium A containing 10 μg/ml insulin; TOV21G and IOSE cells in a 1:1 mixture of Medium 199 (Millipore-Sigma M5017) with sodium bicarbonate (1.1 g/L) and MCDB105 (Millipore-Sigma M63395) with 10% (v/v) FBS; RFT in MEBM medium (Lonza Walkersville, Inc) with MEMB Bullet Kit supplements (excluding gentamycin) and 1% (vol/vol) FBS; JHOC5 cells in DMEM/F12 medium with 10% (v/v) FBS, 0.10 mM nonessential amino acids; and PEO1 cells and their derivatives as well as CaOV3 cells in DMEM medium with 4.5 g/L glucose, 10 μg/ml insulin, 0.04 mM nonessential amino acids, and 10% (vol/vol) FBS. All media contained 50 units/mL penicillin G, 50 μg/mL streptomycin, and 1 mM glutamine.

Drug sensitivity assays

To assess colony formation, aliquots containing 600 IGROV1 or IGROV1/CP cells were plated in medium A in 6 well plates, allowed to adhere for 4 h, and treated with diluent (0.1% DMSO) or varying concentrations of the indicated drugs continuously for 10 days until colonies formed in control plates. Survival was calculated as the ratio of colonies in drug-treated wells compared with diluent-treated wells. Clonogenic assays in other lines were performed with the following changes: i) media listed in the previous paragraph were utilized; ii) the number of cells plated/well was adjusted for COV362 (500 cells), CaOV3 (800), OVCAR3 (2500), A2780 (400), A2780 CP200 (800), TOV21G (500), JHOC5 (500), OVISE (2500) and IOSE cells (300) to assure 150-200 colonies/plate in control plates; and incubation times were extended to 14 days for IOSE and OVCAR3 cells because of their slower doubling time. To assess survival morphologically, cells were treated with the TH588 at 0, 5, or 10 μM for 48 h, after which photographs were taken using an Olympus CKX41 inverted microscope equipped with a 40X UplanFL N Ph2 lens (N.A. 0.75) and a Nikon D90 camera.

Immunoblotting

To prepare whole cell lysates, cells were trypsinized, diluted with medium, sedimented at 200 x g, and washed in calcium- and magnesium-free Dulbecco's phosphate buffered saline (PBS).

PDX tissue was cut to 1 mm sections on dry ice. Samples were sonicated in buffered 6 M guanidine hydrochloride containing 250 mM Tris-HCl (pH 8.5 at 20 °C) and 10 mM EDTA with freshly added 1% (v/v) 2-mercaptoethanol and 1 mM phenylmethylsulfonyl fluoride (PMSF). Samples were reacted with iodoacetamide, dialyzed sequentially into 4 M urea and 0.1% (v/v) SDS, lyophilized, and reconstituted in sample buffer [4 M urea with 2% SDS, 62.5 mM Tris-HCl (pH 6.8), and 0.1% bromophenol blue] as described (4). Aliquots containing 50 µg of protein (assayed by the bicinchoninic acid method) were separated on SDS-polyacrylamide gels containing 8% (wt/vol) acrylamide, transferred to nitrocellulose and probed with antibodies (5).

8-oxo-2'-guanosine (8-oxo-dG) staining

Immunofluorescence assays in tissue culture lines were performed as previously described (6, 7). Cells growing on coverslips were treated as indicated for 24 h, washed in PBS, fixed on ice in 1:1 (vol:vol) methanol:acetone for 20 min, and denatured with 2 N HCl (45 min at RT). Following 3 washes in PBS, cells were neutralized for 10 min with 0.1 M $Na_2B_4O_7$ (pH 8.8), washed in PBS, permeabilized with 0.5% (w/v) Triton X-100 in PBS for 15 min and blocked in 4% (w/v) BSA in PBS for 1 h. Cells were incubated with Abcam anti-8-oxo-dG antibody at 1:200 in 4% BSA in PBS overnight at 4°C. Following five PBS washes, Alexa Fluor 488 secondary antibody (1:1000 in 4% BSA in PBS) was added for 30 min at 37 °C. Finally, after washes in PBS, cells were stained with 1 µg/ml Hoechst 33258 in PBS and mounted with ProLong antifade reagent (Invitrogen; Carlsbad, CA). Images were captured on a LSM 710 scanning confocal microscope (Carl Zeiss AG; Oberkochen, Germany) using a 63X/1.2 W Korr C-Apo objective and processed using Zeiss Zen software.

Immunostaining of PDX specimens was performed as recently described (8). In brief, 4 µm sections of formalin fixed, paraffin embedded (FFPE) samples were deposited onto glass slides. Sections were deparaffinized three times in xylene for 10 min each, sequentially rehydrated twice in 100%, 90%, and 70% ethanol for 3 min each, washed twice in PBS, and permeabilized with 0.2% (w/v) Triton X-100 in PBS for 10 min at 20 °C. After two PBS washes, sections were incubated with 10 µg/mL proteinase K in PBS for 30 min at 37 °C followed by 200 µg/mL RNase A in 10 mM Tris-HCl (pH 7.4), 1 mM EDTA, and 0.4 mM NaCl for 1 h at 37 °C. After three 5-min washes in ddH_2O, slides were treated at 20 °C with 4 N HCl for 25 min, 50 mM Tris base for 15 min, and 10% (v/v) goat serum in PBS for 30 min. Sections were then incubated with Bio-Techne anti-8-oxo-dG diluted at 1:400 in PBS/10% goat serum overnight at 4 °C, washed three times in PBS, incubated with anti-mouse Alexa Fluor 488-conjugated secondary antibody (1:600) for 1 h in the dark at 20 °C, washed once with PBS, incubated with 20 µM Hoechst 33342 for 2 min, and mounted in Prolong Gold Antifade (ThermoFisher). Images were captured with a Zeiss LSM780 confocal microscope equipped with 100x objective.

XRCC1 and γH2A.X foci

A2780 cells were allowed to adhere to 100 mm dishes for 24-48 hours. After a 24 h incubation with 500 nM karonudib, cells were released by brief trypsinization, washed twice with ice cold medium, and suspended at 3 x 10^5 cells/ml so that 100 µl could be sedimented onto slides. Medium was removed and cells were fixed in 4% (w/v) paraformaldehyde for 15 min, permeabilized with 0.25% (w/v) Triton X-100 in PBS, washed five times with wash buffer (PBS containing 0.1% v/v Triton X-100 and 0.1% w/v bovine serum albumin), and blocked overnight with 10% (w/v) powdered nonfat milk in 150 mM NaCl containing 10 mM Tris-HCl (pH 7.4). All steps were performed at 4 °C unless otherwise indicated. Coverslips were incubated with rabbit anti-XRCC1 (1:2000) and mouse monoclonal antibody to γH2AX (1:750) in antibody dilution buffer [5% (v/v) goat serum in PBS] overnight at 4 °C, washed 5 times over 20 min with wash buffer, incubated with secondary antibody (Alexa fluor 488 goat anti-rabbit IgG and Alexa fluor 568 goat anti-mouse IgG, each at 1:1000) in antibody dilution buffer for 1 h at 21 °C in the dark,

washed six times with wash buffer, counterstained with 1 μg/ml Hoechst 33258 in PBS, and mounted with Prolong anti-fade mounting fluid. Cells were examined using a Zeiss Axio Observer microscope equipped with a 63X NA 1.4 lens, and images were captured with a Zeiss Axiocam 305 color camera using Zeiss Zen Pro software. From each of three independent experiments, 100 cells were imaged and scored in a blinded manner.

Assays for mitotic cells

Cells were assayed for the mitotic marker phospho-Ser^{28}-Histone H3 by flow cytometry as previously described (9). In brief, after cells were treated with paclitaxel, TH588 or karonudib for 24 h, adherent and floating cells were collected, pooled, washed, and fixed in 70% ethanol at -20 ^{o}C for 4 hrs. Cells were then stained with Alexa Fluor 647-conjugated rat anti-phospho-Ser^{28}-Histone H3 antibody, incubated with 50 μg RNaseA at 37 ^{o}C, and stained with propidium iodide (10 μg/ml). Flow cytometry was performed on a FACSCanto II flow cytometer using the FL2 (excitation 488 nm; emission 585/42 nm) and FL4 channels (excitation 633 nm; emission 660/20 nm). After 20,000 events were collected, data were analyzed with BD CellQuest software.

Alternatively, mitotic cells were visualized by fluorescence microscopy after staining with Hoechst 33258 (10). In brief, drug-treated cells were washed, allowed to swell in 75 mM KCl for 15 min at 37 °C, sedimented at 50 x g, fixed in a 3:1 (vol:vol) solution of methanol:acetic acid for 1 h at 20 °C, pelleted, dropped onto slides, allowed to air dry, and stained with 1 μg/mL Hoechst 33258 in 50% glycerol/50% 0.1 M Tris-HCl (pH 7.4).

PDX treatment *in vivo*

Under the aegis of protocols approved by the Mayo Clinic Institutional Review Board and Mayo Clinic Animal Care and Use Committee, HGSOC PDXs were established as intraperitoneal tumorgrafts and monitored twice weekly by transabdominal ultrasound to determine the largest detectable tumor cross-section area as reported earlier (11, 12, 13). These PDXs, which were also previously subjected to targeted capture and massively parallel sequencing of a panel of ovarian cancer susceptibility genes (11), were allowed to grow to a tumor area of 0.5-0.8 cm^2. Mice were then randomized to one of four treatment arms: Diluent (n = 8), carboplatin (50 mg/kg i.p. on day 1 of each seven-day cycle; n = 8), karonudib (90 mg/kg by oral gavage twice daily on days 2-4; n = 10), or the combination [carboplatin 50 mg/kg i.p. on day 1 plus karonudib 90 mg/kg (PH384) or 60 mg/kg (PH013 and PH450) by gavage on days 2-4; n = 12]. The dose and schedule were based on a prior study showing that karonudib up to 90 mg/kg as monotherapy by oral gavage was tolerable (2). That study also showed that karonudib, which has an oral bioavailability of >60%, a V_d of ~3 L/kg, and a serum half-life of ~4 h, achieves a C_{max} of ~7 μM following administration of 50 mg/kg to NOD-SCID mice by oral gavage.

Tumors were harvested from 1-2 animals/treatment arm for analysis of 8-oxo-dG staining on Day 5. After four treatment cycles were completed, mice were observed without further treatment until tumors recurred or mice met humane endpoints (14). All experiments conformed to the approved Animal Care and Use Committee protocol; and no tumors were allowed to grow beyond the approved diameter of 1.7 cm as assessed by transabdominal ultrasound once or twice weekly.

mRNA Sequencing

A2780 cells treated with 0.1% (v/v) DMSO or 6 μM TH588 for 48 hours were harvested in biological triplicates for mRNA sequencing. RNA was purified using a Qiagen RNeasy kit. Residual DNA was removed by on-column digestion using the RNase-free DNAase according to the supplier's instructions. RNA sample quality was assessed by RNA integration number (RIN) on the RNA ScreenTape System (Agilent) according to manufacturer's protocol. cDNA was prepared with the TruSeq RNA sample prep kit v2 (Illumina). RNA-seq libraries were prepared from 100-200 ng high-quality RNA using TruSeq RNA Library Prep Kit v2 (Illumina) and analyzed

on an Illumina HiSeq 4000.

Bioinformatic analysis and qRT-PCR validation of mRNA sequencing
Raw RNA sequencing reads were aligned to the UCSC human genome (GRCh38) using TopHat (v2.0.14) (15). Gene level counts were obtained using featureCounts 1.4.6 from the subRead package with gene models from corresponding UCSC annotation packages. Differential expression analysis was performed in R using DESeq2. Figures were generated using ComplexHeatmap (16).

For qRT-PCR, RNA was isolated from 50-60% confluent cultures of A2780 cells treated with diluent, 6 μM TH588 or 500 nM karonudib for 72 h beginning on 4-5 separate days. qRT-PCR was performed in duplicate using 100 ng RNA and TaqMan RNA-to-C_T 1-Step Kit (Applied Biosystems, Carlsbad, CA) per the supplier's instructions. Using probesets listed in **Table S2**, PCR was performed on a CFX384 Real Time System (C10000 Touch Thermal Cycler, BioRad, Hercules, CA) using a program consisting of 48 °C for 15 min, 95 °C for 10 min, then 40 cycles of 95 °C for 15 sec and 60 °C for 1 min. Data were analyzed using the following equations: $\Delta\Delta C_t$=ΔC_t(sample)- ΔC_t(endogenous control); and Fold Change = $2^{-\Delta\Delta Ct}$, and assessed for relative change as indicated in various figures.

Statistical Analysis
Colony forming assays were performed independently at least three times unless otherwise indicated. After normalization to diluent-treated controls, mean survival results from multiple assays were plotted as the mean ± SEM survival at each drug concentration. IC_{50} values from individual experiments were determined by fitting each log-linear curve using a quadratic function in Prism v. 10.4.1 and determining the intercept of the curve with % colony formation = 50%.

For PDX tumor growth analysis, linear mixed effects modeling, performed in the SAS PROC MIXED procedure, was used to assess differences between groups (17). Tumor area on the natural log scale was compared between treatments using a growth model framework. The time variable was centered for hypothesis testing. Because of occasional differences in measurement intervals, a spatial power correlation structure, which assumes any two observations from the same mouse are correlated and that this correlation decreases exponentially with time between the observations, was specified in the REPEATED statement. For visualization, model estimates with 95% confidence intervals were plotted for each treatment group, scaled to the baseline average. Two degree-of-freedom contrasts were performed to test for coincident curves, i.e., to assess simultaneous difference in slope and intercept between treatment trajectories. Mouse overall survival, defined as the time from treatment initiation (Day 0) to animal death or unplanned sacrifice, is shown via Kaplan Meier curves. Cox proportional hazards regression models were used to estimate hazard ratio (HR) and p value.

SUPPLEMENTARY REFERENCES

1. Gad H, Koolmeister T, Jemth AS, Eshtad S, Jacques SA, Strom CE, et al. MTH1 inhibition eradicates cancer by preventing sanitation of the dNTP pool. Nature. 2014;508(7495):215-21.
2. Warpman Berglund U, Sanjiv K, Gad H, Kalderen C, Koolmeister T, Pham T, et al. Validation and development of MTH1 inhibitors for treatment of cancer. Ann Oncol. 2016;27(12):2275-83.
3. Wahner Hendrickson AE, Visscher DW, Hou X, Goergen KM, Atkinson HJ, Beito TG, et al. CHFR and Paclitaxel Sensitivity of Ovarian Cancer. Cancers (Basel). 2021;13(23).
4. Kaufmann SH, Svingen PA, Gore SD, Armstrong DK, Cheng Y-C, Rowinsky EK. Altered Formation of Topotecan-Stabilized Topoisomerase I-DNA Adducts in Human Leukemia Cells. Blood. 1997;89:2098-104.
5. Kaufmann SH. Reutilization of Immunoblots After Chemiluminescent Detection. Analytical Biochemistry. 2001;296:283-6.
6. Patel A, Burton DG, Halvorsen K, Balkan W, Reiner T, Perez-Stable C, et al. MutT Homolog 1 (MTH1) maintains multiple KRAS-driven pro-malignant pathways. Oncogene. 2015;34(20):2586-96.
7. Rai P, Young JJ, Burton DG, Giribaldi MG, Onder TT, Weinberg RA. Enhanced elimination of oxidized guanine nucleotides inhibits oncogenic RAS-induced DNA damage and premature senescence. Oncogene. 2011;30(12):1489-96.
8. Kanakkanthara A, Hou X, Ekstrom TL, Zanfagnin V, Huehls AM, Kelly RL, et al. Repurposing Ceritinib Induces DNA Damage and Enhances PARP Inhibitor Responses in High-Grade Serous Ovarian Carcinoma. Cancer Res. 2022;82(2):307-19.
9. Ding H, Vincelette ND, McGehee CD, Kohorst MA, Koh BD, Venkatachalam A, et al. CDK2-Mediated Upregulation of TNFalpha as a Mechanism of Selective Cytotoxicity in Acute Leukemia. Cancer Res. 2021;81(10):2666-78.
10. Blajeski AL, Kottke TJ, Kaufmann SH. A multistep model for paclitaxel-induced apoptosis in human breast cancer cell lines. Exp Cell Res. 2001;270(2):277-88.
11. AlHilli MM, Becker MA, Weroha SJ, Flatten KS, Hurley RM, Harrell MI, et al. In vivo anti-tumor activity of the PARP inhibitor niraparib in homologous recombination deficient and proficient ovarian carcinoma. Gynecol Oncol. 2016;143(2):379-88.
12. Weroha SJ, Becker MA, Enderica-Gonzalez S, Harrington SC, Oberg AL, Maurer MJ, et al. Tumorgrafts as in vivo surrogates for women with ovarian cancer. Clin Cancer Res. 2014;20:1288-97.
13. Hurley RM, McGehee CD, Nesic K, Correia C, Weiskittel TM, Kelly RL, et al. Characterization of a RAD51C-Silenced High Grade Serous Ovarian Cancer Model During Development of PARP Inhibitor Resistance. NAR Cancer. 2021;3:zcab028.
14. Paster EV, Villines KA, Hickman DL. Endpoints for mouse abdominal tumor models: refinement of current criteria. Comp Med. 2009;59(3):234-41.
15. Kim D, Pertea G, Trapnell C, Pimentel H, Kelley R, Salzberg SL. TopHat2: accurate alignment of transcriptomes in the presence of insertions, deletions and gene fusions. Genome Biol. 2013;14(4):R36.
16. Gu Z, Eils R, Schlesner M. Complex heatmaps reveal patterns and correlations in multidimensional genomic data. Bioinformatics. 2016;32(18):2847-9.
17. Oberg AL, Heinzen EP, Hou X, Al Hilli MM, Hurley RM, Wahner Hendrickson AE, et al. Statistical analysis of comparative tumor growth repeated measures experiments in the ovarian cancer patient derived xenograft (PDX) setting. Sci Rep. 2021;11(1):8076.
18. Stordal B, Farrelly AM, Hennessy BT. Chromosomal copy number and mutational status are required to authenticate ovarian cancer cell lines as appropriate cell models. Mol Biol Rep. 2024;51(1):784.

19. Anglesio MS, Wiegand KC, Melnyk N, Chow C, Salamanca C, Prentice LM, et al. Type-specific cell line models for type-specific ovarian cancer research. PLoS One. 2013;8(9):e72162.
20. Al Hilli MM, Becker MA, Weroha SJ, Flatten KS, Hurley RM, Harrell MI, et al. In vivo anti-tumor activity of the PARP inhibitor niraparib in homologous recombination deficient and proficient ovarian carcinoma. Gynecol Oncol. 2016;143:379-88.
21. Kaufmann T, Tai L, Ekert PG, Huang DC, Norris F, Lindemann RK, et al. The BH3-only protein bid is dispensable for DNA damage- and replicative stress-induced apoptosis or cell-cycle arrest. Cell. 2007;129(2):423-33.
22. Friedman HS, Dolan ME, Kaufmann SH, Colvin OM, Griffith OW, Moschel RC, et al. Elevated DNA Polymerasea, DNA Polymeraseb, and DNA Topoisomerase II in a Melphalan-Resistant Rhabdomyosarcoma Xenograft that is Cross-Resistant to Nitrosoureas and Topotecan. Cancer Research. 1994;54:3487-93.
23. Sanchez ER, Toft DO, Schlesinger MJ, Pratt WB. Evidence that the 90-kDa phosphoprotein associated with the untransformed L-cell glucocorticoid receptor is a murine heat shock protein. J Biol Chem. 1985;260(23):12398-401.
24. Caserta TM, Smith AN, Gultice AD, Reedy MA, Brown TL. Q-VD-OPh, a broad spectrum caspase inhibitor with potent antiapoptotic properties. Apoptosis. 2003;8(4):345-52.
25. Kaufmann SH, Desnoyers S, Ottaviano Y, Davidson NE, Poirier GG. Specific Proteolytic Fragmentation of Poly(ADP-ribose) Polymerase: An Early Marker of Chemotherapy-Induced Apoptosis. Cancer Research. 1993;53:3976-85.
26. Lazebnik YA, Kaufmann SH, Desnoyers S, Poirier GG, Earnshaw WC. Cleavage of Poly(ADP-ribose)Polymerase by a Proteinase with Properties like ICE. Nature. 1994;371:346-7.
27. Rudd SG, Gad H, Sanjiv K, Amaral N, Hagenkort A, Groth P, et al. MTH1 Inhibitor TH588 Disturbs Mitotic Progression and Induces Mitosis-Dependent Accumulation of Genomic 8-oxodG. Cancer Res. 2020;80(17):3530-41.

**Table S1<br>Histological and Genomic Characteristics of Cell Lines and PDXs**		
**Cell lines**		
Cell line	Likely histologic subtype	Major reported oncogenic alterations[a]
A2780	Endometrioid	PIK3CA E365K, PTEN K128_R130del, ARID1A Q1430*
CAOV3	HGSOC	TP53 Q136*, ATM S1905Ifs*25
COV362	HGSOC	TP53 Y220C, BRCA1 X1366_splice
IGROV1	HGSOC (18)	TP53 S90Lfs*59, TP53 Y126C, BRCA1 K654Sfs*47, BRCA2 K1108Rfs*11, PIK3CA R38C, PTEN T319fs*1, PTEN V317fs*3, RB1 V654Cfs*4
PEO1	HGSOC	TP53 G244D, BRCA2 Y1655L, CREBBP E1238*
JHOC5	Clear cell (19)	ARID1A G122Afs*110
TOV21G	Clear cell (19)	KRAS G13C, PIK3CA H1047Y, ARID1A Y551Lfs*72 and Q758Rfs*75, PTEN L267Rfs*9
OVISE	Clear cell (19)	PIK3CA C420R, ARID1A Q542Pfs*80 and H203Qfs*197
**PDXs**		
PDX	Histologic subtype	Mutations[b]
PH013	HGSOC	TP53 p.T253P
PH384	HGSOC	TP53 c.501delG; LIG4 p.Q787X
PH450	HGSOC	CHEK2 c1100delC

[a]Information from https://sites.broadinstitute.org/ccle/ and https://www.cellosaurus.org/index.html
[b]Mutation analysis in PDX models was performed by BROCA-HR as reported (20).

**Table S2<br>Sources of Reagents**			
**Reagents**			
Reagent	Supplier	Location	Catalog number
Bovine serum albumin	Millipore-Sigma	St. Louis, MO	A7906
Carboplatin	Mylan	Canonsburg, PA	N.A.
CM-H_2DCFDA	ThermoFisher	Waltham, MA	C6827
Cisplatin	Millipore-Sigma	St. Louis, MO	P4394
Hoechst 33258	Millipore-Sigma	St. Louis, MO	B2883
Paclitaxel injection	Sagent	Schaumburg, IL	N.A.
Paclitaxel powder	Millipore-Sigma	St. Louis, MO	T7402
Q-VD-OPh	SM Biochemicals	Anaheim, CA	SMPH001
**Antibodies**			
Target antigen	Supplier	Antibody type	Product number or citation
BAK	Cell Signaling Technology	Rabbit mAb	12105S
BAX	Cell Signaling Technology	Rabbit mAb	2774S
BBC3 (Puma)	Santa Cruz Biotechnology	Mouse mAb	sc-374223
BCL2	Dako	Mouse mAb clone 124	M0887
BCL2L1 (BCLW)	Cell Signaling Technology	Rabbit mAb	2724S
BCL2L2 (BCLXL)	Cell Signaling Technology	Rabbit mAb	2964S
BCL2L11 (BIM)	Cell Signaling Technology	Rabbit mAb	2933S
BMF	Cell Signaling Technology	Rabbit mAb	50542S
BID	David Huang, WEHI, Melbourne Australia	Rat mAb clone AE6	(9, 21)
BRCA1	Cell Signaling Technology	Rabbit Ab	9010S
BRCA2	Cell Signaling Technology	Rabbit mAb	10741S
CDKN1A (p21)	EMD Millipore	Mouse mAb clone CP74	05-655
CHFR		Mouse mAb	(3)
CYC	Becton Dickinson	Mouse mAb	7H8.2C12
GAPDH	Cell Signaling Technology	Rabbit mAb	2118S
GST pi	Biotrin International	Rabbit Ab	(9, 22)
Heat shock protein 90β (clone H-90-10)		Mouse mAb	(23)
Phospho-Ser^{139} Histone H2A.X	EMD Millipore	Mouse mAb clone JBW301	05-637

Histone H2A.X	Cell Signaling Technology	Rabbit Ab	2595S
Phospho-Ser^{28}-Histone H3	Upstate	Rabbit antibody	06-570
Phospho-Ser^{28}-Histone H3, Alexa Fluor 647 labeled	BD Bioscience	Rat antibody	558217
HRK	ProSci	Rabbit Ab	3771
8-Hydroxy-2’-deoxyguanosine	Abcam	Mouse mAb	ab48508
Lamin B1	Santa Cruz Biotechnology	Mouse mAb	sc-377000
MCL1	Cell Signaling Technology	Rabbit Ab	4272S
MTH1	Abcam	Rabbit mAb	ab200832
8-oxo-2’-deoxyguanosine	BioTechne	Mouse mAb	4354-MC-50
RAD51	Neomarkers	Mouse mAb	Ms-988-P
RAF	Santa Cruz Biotechnology	Rabbit Ab	Sc-133
TP53	Invitrogen	Mouse mAb clone 1801	MA 5-11296
XRCC1	Cell Signaling Technology	Rabbit Ab	2735S

qRT-PCR probes

Target transcript	Supplier	Product number or citation
ACTB	Applied Biosystems	4332645
BAX	Applied Biosystems	4331182 (Hs00180269_m1)
BBC3 (PUMA)	Applied Biosystems	4331182 (Hs00248075_m1)
BCL2	Applied Biosystems	4331182 (Hs00608023_m1)
BCL2L2 (BCLXL)	Applied Biosystems	4331182 (Hs00708019_m1)
BCL2L11 (BIM)	Applied Biosystems	4331182 (Hs00236329_m1)
BLM	Applied Biosystems	4331182 (Hs00172060_m1)
BMF	Applied Biosystems	4331182 (Hs05050813_m1)
BRCA1	Applied Biosystems	4331182 (Hs01556193_m1)
BRCA2	Applied Biosystems	4331182 (Hs00609073_m1)
DCLRE1A	Applied Biosystems	4331182 (Hs00384872_m1)
FANCD2	Applied Biosystems	4331182 (Hs00276992_m1)
GADD45A	Applied Biosystems	4331182 (Hs00169255_m1)
MCL1	Applied Biosystems	4331182 (Hs03043898_m1)
PMAIP1 (NOXA)	Applied Biosystems	4331182 (Hs00560402_m1)
POLQ	Applied Biosystems	4331182 (Hs00981375_m1)
RPLPO	Applied Biosystems	4331182 (Hs00420895_m1)
RRM2B	Applied Biosystems	4331182 (Hs00968432_m1)

Abbreviations: N.A., not applicable.

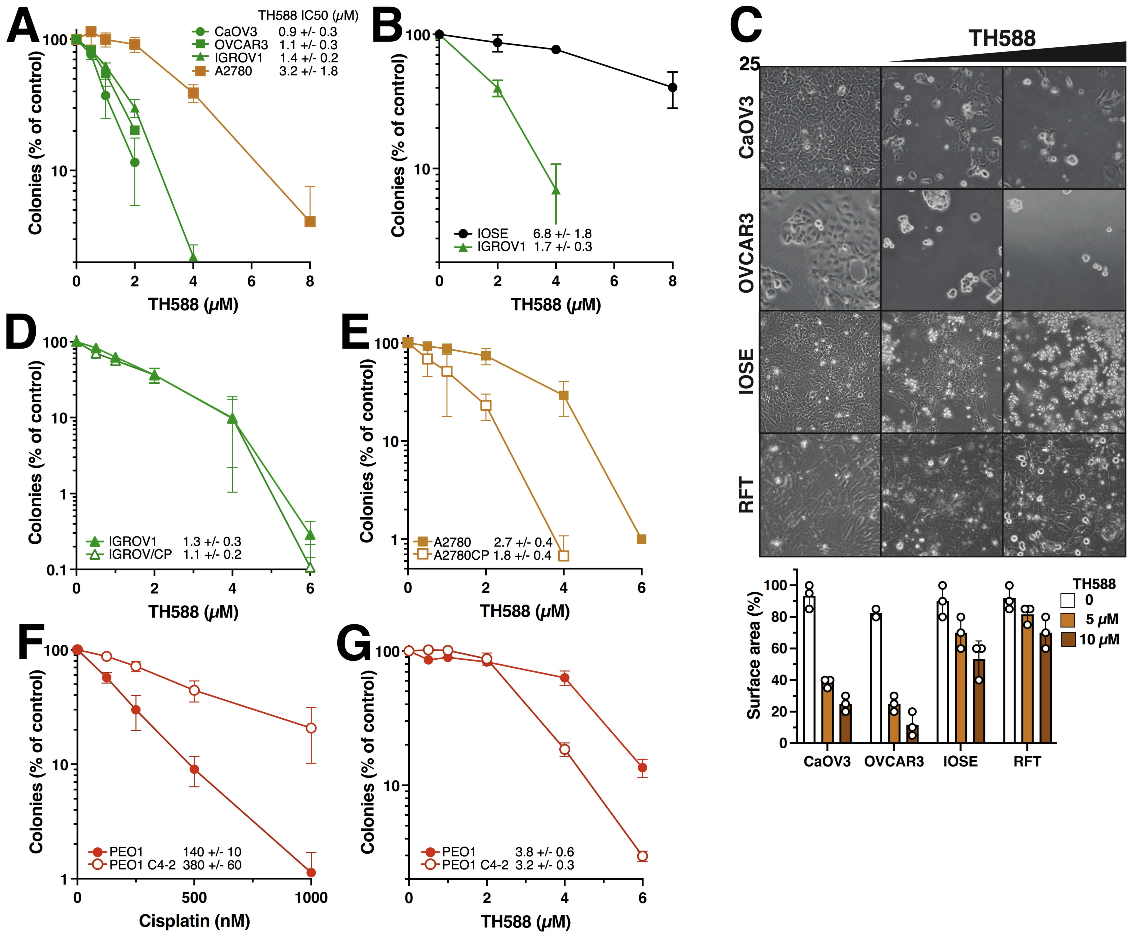

Figure S1, related to Figure 1. Effect of mitotic MTH1 inhibitor (mMTH1i) TH588 on ovarian cancer cell lines and nontransformed cells. A, B, OC cell lines CaOV3, OVCAR3, A2780, and IGROV1 (A) or IGROV1 and immortalized normal ovarian surface epithelial cells (IOSE, B) were treated continuously with the mMTH1i TH588 in clonogenic assays. **C,** phenotypic assay for TH588 sensitivity. Because nontransformed resected fallopian tube epithelial cells (RTF) do not form colonies, two OC cell lines (CaOV3 and Ovcar3) as well as IOSE and RFT cells were photographed after exposure to 0, 5 or 10 µM TH588 for 48 h. Graph below micrographs shows percentage of surface area covered by cells at the 48 h time point. Error bars, mean ± SEM of 3 independent experiments. **D-G,** paired parental IGROV1 and platinum-selected IGROV1/CP cells (D) or A2780 and platinum-selected A2780/CP200 cells (E), or PE01 and platinum-selected PEO1 C4-2 (F, G) were treated continuously with the indicated concentrations of TH588 (D, E, G) or cisplatin (F). All results are mean ± SEM of ≥3 independent experiments except IOSE cells, which were assayed twice independently. IC_{50} values for parental A2780 and IGROV1 cells vary slightly between panels because resistant lines were directly compared to parental lines, which were repeated for those experiments. Platinum and karonudib sensitivities of IGROV1/CP and A2780/CP200 cells are shown in Figure 1.

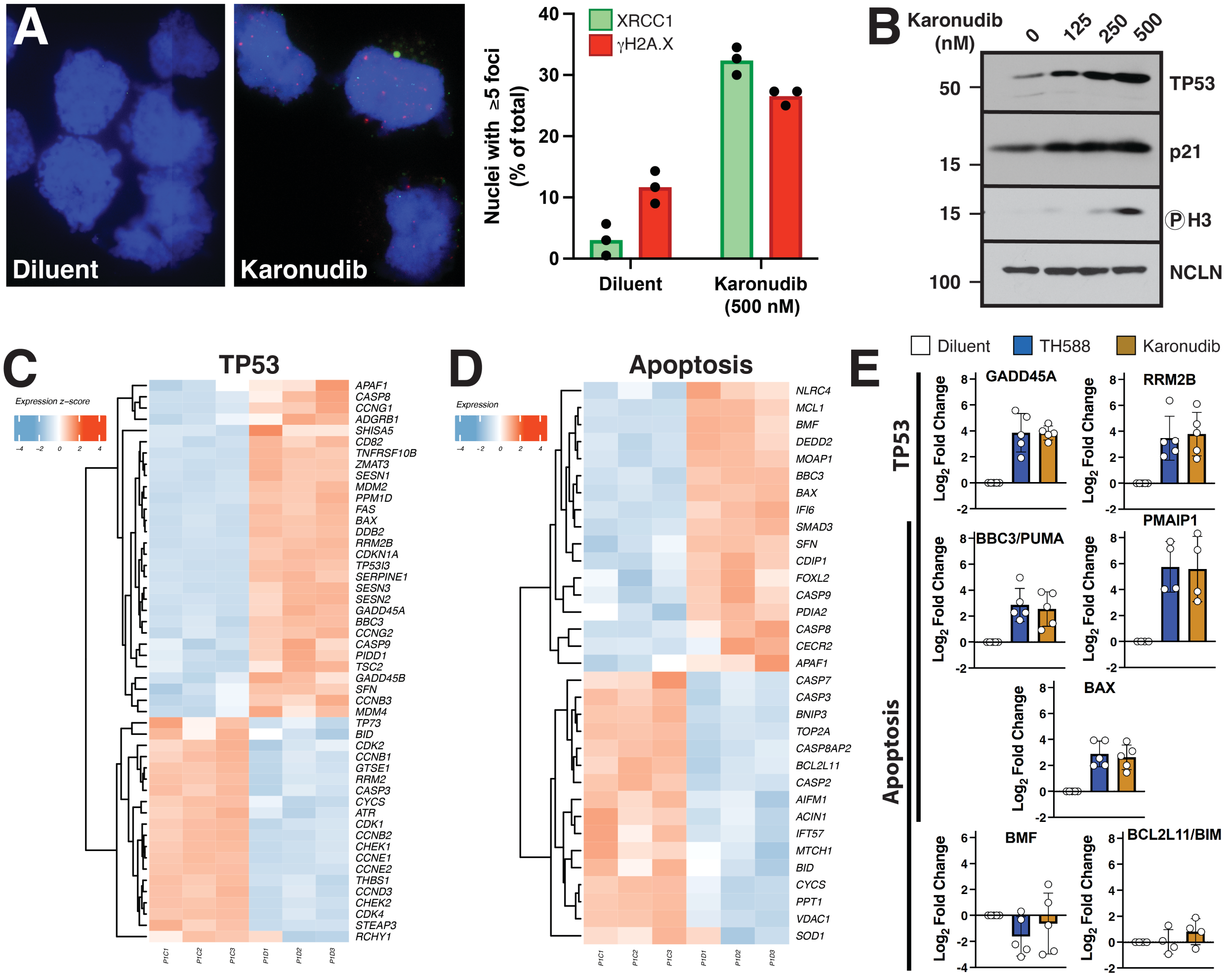

Figure S2 (related to Figure 1). Cellular effects of mMTH1 inhibitor treatment. A, activation of base excision repair. A2780 cells were treated for 48 h with diluent (0.1% DMSO) or 500 nM karonudib and stained for XRCC1 foci (green, an indicator of ongoing base excision repair) and γH2A.X foci (red, an indicator of ATM, ATR or DNA-PK activation). Results from three independent assays are shown in the bar graph. **B, upregulation of TP53, p21 and histone H3 phosphorylation.** After A2780 cells were treated with diluent or the indicated concentration of karonudib for 48 h, whole cell lysates were subjected to SDS-polyacrylamide gel electrophoresis and immunoblotting for the indicated antigen. As previously reported (2), TP53 and p21 (the product of the TP53 target gene *CDKN1A*) were upregulated. **C-E, effects of mMTH1 treatment on gene expression.** A2780 cells were treated for 48 h with 6 µM TH588 (C, D) and subjected to RNA sequencing as described in the Supplementary Methods. Changes in transcript levels in the KEGG TP53 pathway and apoptosis pathway are shown. Alternatively, A2780 cells were treated for 72 h with 6 µM TH588 or 500 nM karonudib and subjected to qRT-PCR to confirm the results of the RNA sequencing (E). At the mRNA level, upregulation of a number of TP53 regulated transcripts , including transcripts encoding the pro-apoptotic proteins BAX, BBC3/Puma, and PMAIP1/Noxa, was observed as well as modulation of additional BCL2 family members. Notably, TH588 and karonudib had similar effects in A2780 cells at the mRNA level.

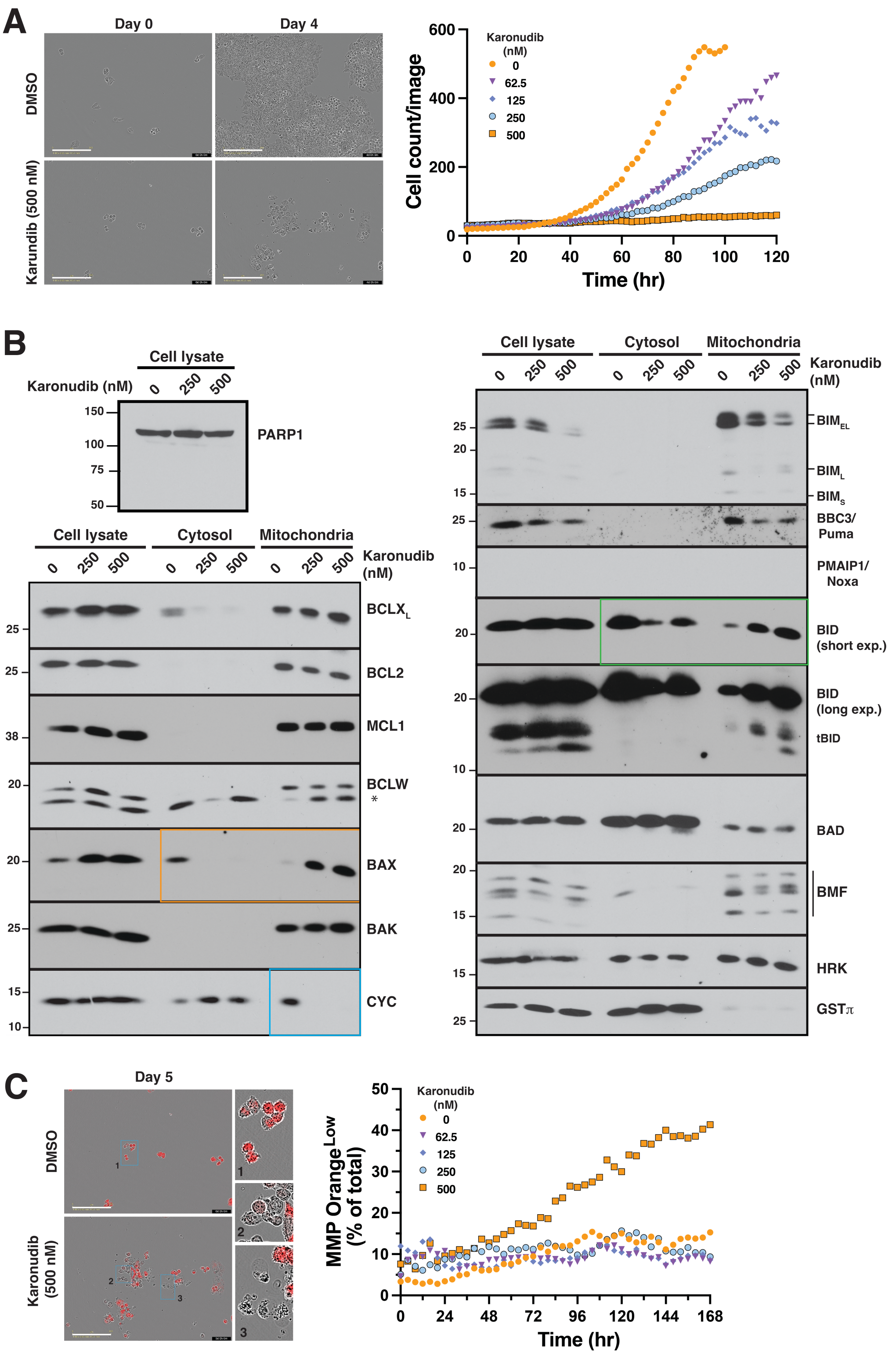

Figure S3 (related to Figure 1). Antiproliferative and cytotoxic effects of karonudib in A2780 cells. A, antiproliferative effects revealed by live cell imaging. A2780 cells were treated with the indicated concentrations of karonudib over a 120-hour period and quantitated using the AI module in a Sartorius Sx5 Incucycte live cell image analysis system. Left panels show screen shots at 0 and 96 h in the presence of diluent (0.1% DMSO) or 500 nM karonudib. Bar indicates

200 µm. Right panel shows mean results from 4 images from each of 2 identical wells in one of two experiments performed in this fashion. **B, induction and mitochondrial translocation of BAX during karonudib treatment.** After A2780 cells were treated for 72 hours with diluent or the indicated concentrations of karonudib in the presence of the broad spectrum caspase inhibitor Q-VD-OPh (24) at 5 µM, cell lysates as well as mitochondrial and cytosolic fractions were subjected to SDS-PAGE and immunoblotting for the indicated antigens. Upper left, blotting of whole cell lysates for PARP1 reveals intact protein and no detectable generation of the caspase-generated 85 kDa fragment (25, 26), indicating that caspases 3 and 7 have been successfully inhibited. Blotting for multi-domain BCL2 family members (lower left) and BH3-only family members (lower right) indicates that, in contrast to results observed at the RNA level (**Figure S2**), at the protein level there was no increase in BBC3/Puma, BMF or MCL1. PMAIP1/Noxa, the product of another TP53 target gene, was not detectable in A2780 cells. In contrast, BAX was translocated from the cytoplasm to the nucleus (orange box), as was a portion of both full-length and truncated forms of the BID protein (green box). Cytochrome c (CYC) was concomitantly released from mitochondria (blue box). GSTπ, a cytoplasmic protein, is shown to confirm efficacy of the fractionation. * indicates nonspecific band. **C, loss of mitochondrial membrane potential and development of apoptotic morphology.** A2780 cells stained with MMP Orange, a dye accumulated in mitochondrial with an intact mitochondrial transmembrane potential (https://www.sartorius.com/download/879808/incucyte-mmp-orange-reagent-product-guide-en-8000-0731-a00-l-1--data.pdf), were treated with the indicated concentrations of karonudib over a 168-hour period in the absence of Q-VD-OPh and visualized in a Sartorius Sx5 Incucycte live cell image analysis system. Left panels show screen shots at 0 and 120 h in the presence of 500 nM karonudib. Blue rectangles numbered 1-3 on thumbnails correspond to higher magnification micrographs 1-3, which contain cells with healthy mitochondria (1), cells with depolarized mitochondria that have not yet developed apoptotic morphological changes (2) and cells with depolarized mitochondria that have developed membrane blebbing, a common morphological feature of apoptotic cells in tissue culture (3). Right panel shows mean results from 4 images from each of 2 identical wells as in panel A. Bar indicates 200 µm.

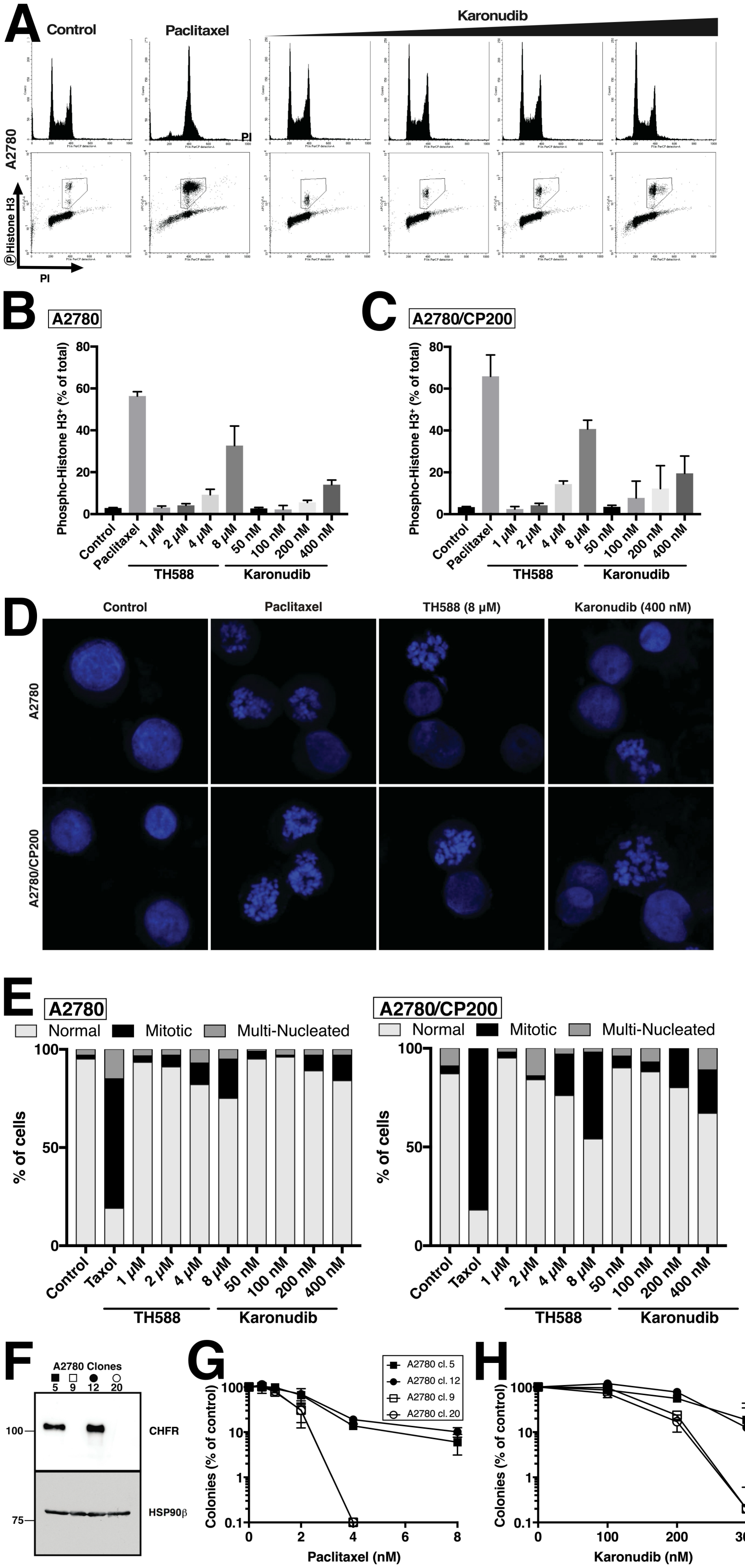

Figure S4. Mitotic stalling by the MTH1is TH588 and karonudib. A-C, A2780 (A, B) and A2780/CP200 (C) were treated with paclitaxel or increasing concentrations of TH588 or karonudib

for 24 h and stained for phospho-Ser^{28}-Histone H3 and PI. **D, E,** Hoechst 33258 staining following paclitaxel, TH588, or karonudib treatment cells is illustrated (D) and summarized for A2780 and A2780/CP200 cells (E). **F-H,** after transfection of A2780 cells with plasmid containing the CHFR open reading frame, clones expressing (closed symbols) or not expressing CHFR (open symbols) were isolated and examined by immunoblotting (F) or subjected to colony forming assays performed using continuous exposure to paclitaxel (G) or karonudib (H). These clones were denoted clones 3,1,4 and 2, respectively, in Supplementary ref. 3.

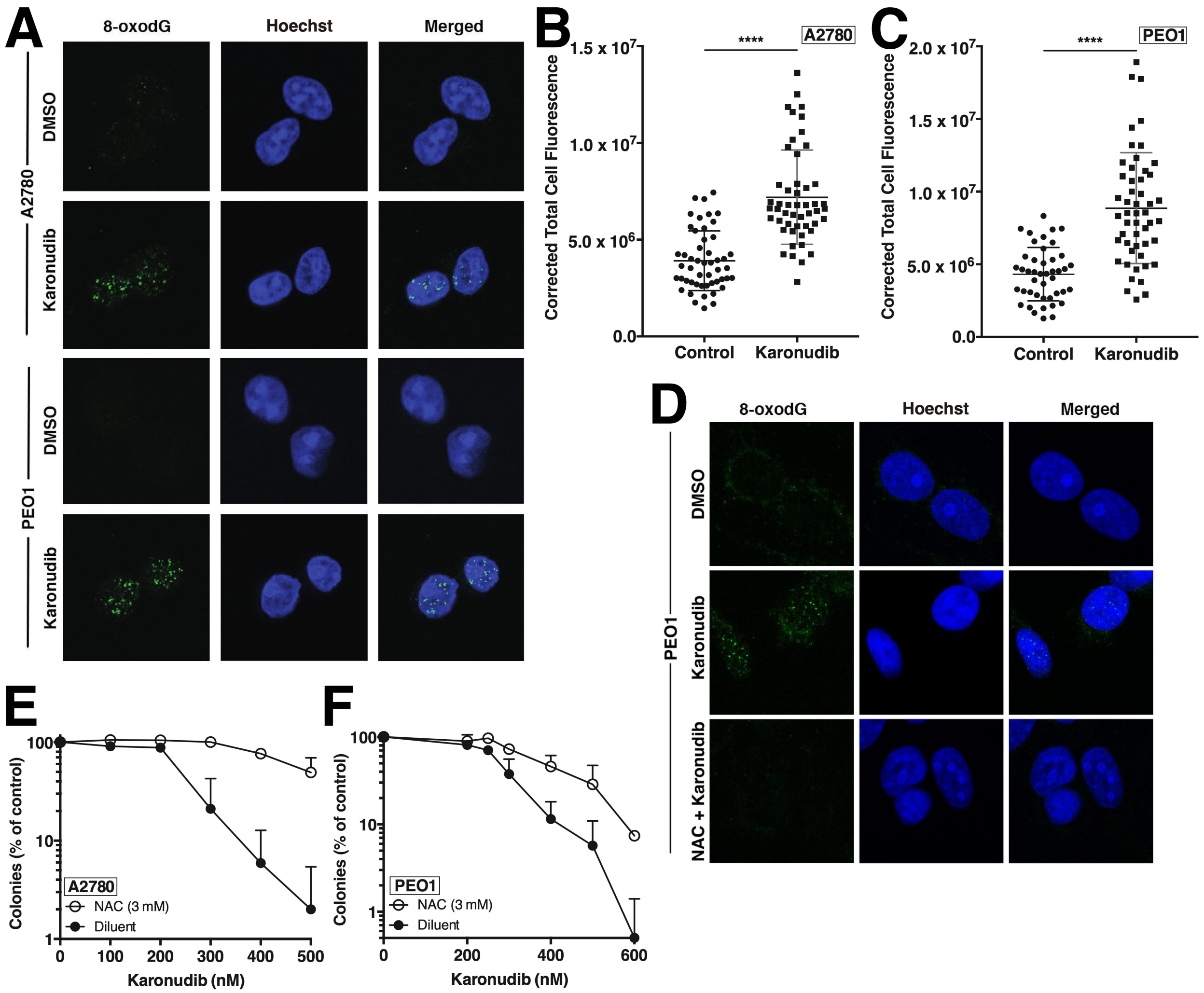

Figure S5. Effect of karonudib and N-acetylcysteine (NAC) on levels of nuclear 8-oxo-dG and colony formation. A-C, following treatment with karonudib, 8-oxo-dG was visualized by immunofluorescence (A) and quantitated in 100 cells/treatment for A2780 (B) and PEO1 (C) cells. **D-F,** following treatment with 3 mM NAC for 4 h to scavenge cellular ROS, cells were treated with 400 nM karonudib and stained for 8-oxo-dG (D) or exposed to varying concentrations of karonudib for 24 h, washed, and allowed to form colonies (E, F). **** in panel C indicates $p<0.0001$ by unpaired t-test. The present results, which provide biochemical confirmation that 8-oxo-dG is required for cytotoxicity of mMTH1is, complement and extend previous chemical and molecular biological results addressing this question (2, 27).

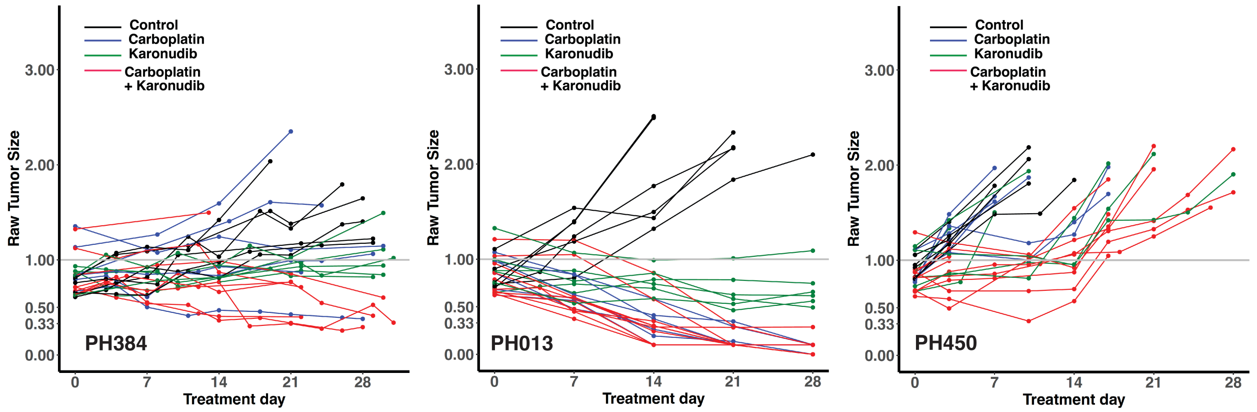

Figure S6 (related to Figure 2). Growth trajectories of PDXs in individual animals as determined by transabominal ultrasound.

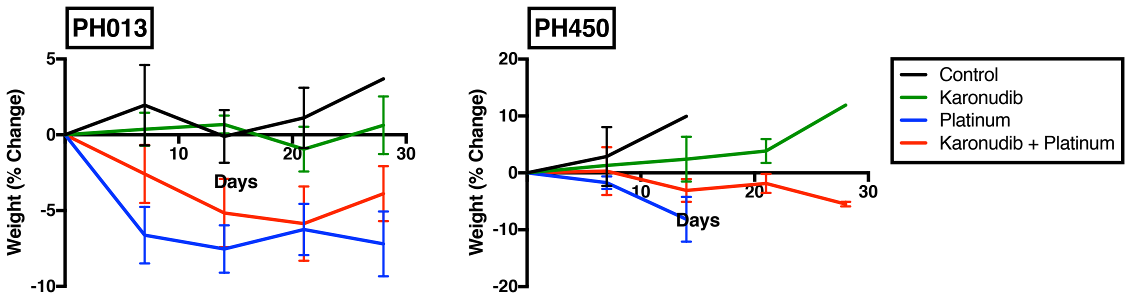

Figure S7 (related to Figure 2). Tolerability of karonudib as a single agent and in combination with carboplatin. In an experiment in PH384, the karonudib dose was decreased from 90 mg/kg to 60 mg/kg in the combination arm due to weight loss in the first week of treatment. Thereafter, animals bearing OC PDX models PH013 (left) or PH450 (right) were treated with karonudib (60 mg/kg bid on days 2, 4 and 6) administered as a single agent or in combination with carboplatin (50 mg/kg/wk on day 1) for four weeks and weighed weekly.
